# The influence of various chemical oxygen demands on microbial fuel cells performance using leachate as a substrate

**DOI:** 10.1007/s11356-024-32090-x

**Published:** 2024-01-29

**Authors:** Aliyu Ishaq, Mohd Ismid Mohd Said, Shamila Binti Azman, Mohammed Rajab Houmsi, Abubakar Sadiq Isah, Zainab Toyin Jagun, Shamsuddeen Jumande Mohammad, Al−Amin Danladi Bello, Umar Alfa Abubakar

**Affiliations:** 1 Department of Water & Environmental Engineering, School of Civil Engineering, Faculty of Engineering, Universiti Teknologi Malaysia, 81300 Bahru, Johor Malaysia; 2 Department of Water Resources and Environmental Engineering, Ahmadu Bello University, Kaduna, 1045 Zaria Nigeria; 3 New Era and Development in Civil Engineering Research Group, Scientific Research Center, AlAyen University, Thi−Qar, Nasiriyah, 64001 Iraq; 4 Department of Real Estate, School of Built Environment Engineering And Computing, Leeds Beckett University, City Campus, Leeds, UK; 5 School of Engineering, Technology, and Design, Canterbury Christ Church University, North Holmes Road, Canterbury, Kent CT1 1QU UK

**Keywords:** Microbial fuel cell, Leachate treatment, Chemical oxygen demand, Landfill leachate, Dual compartment MFC

## Abstract

Microbial fuel cells (MFCs), hailed as a promising technology, hold the potential to combat various wastewater pollutants while simultaneously converting their chemical energy into electricity through biocatalysts. This study explores the applicability of a dual compartment MFC (DC-MFC) under varying conditions, targeting the removal of chemical oxygen demand (COD) from landfill leachate and electricity generation. In this setup, anaerobic sludge from a wastewater treatment plant serves as the inoculum in the anode compartment of the MFC, with a Nafion117 membrane acting as the separator between MFC units. The cathode compartments are filled with distilled water and continually aerated for 24 h to enhance air supply. The study assesses the MFC’s performance across different COD concentrations, focusing on COD removal, power generation, and Coulombic efficiency. The findings reveal that COD removal efficiency is notably enhanced at higher concentrations of organic matter. Specifically, at a COD concentration of 3325.0 mg L^−1^, the MFC exhibited the highest COD removal efficiency (89%) and maximum power density (339.41 mWm^−2^), accompanied by a Coulombic efficiency of 25.5%. However, as the initial substrate concentration increased to 3825 mg L^−1^, the efficiency decreased to 72%, with a Coulombic efficiency of 13.56% and a power density of 262.34 mWm^−2^. Optical density levels increased due to bacterial growth at ambient temperature and neutral pH, reflecting the dynamic microbial response within the system.

## Introductions

In recent years, there has been an increasing scholarly focus on the utilisation of alternate renewable energy sources that possess both environmental friendliness and economic sustainability. The utilisation of bioelectrochemical system technologies, which can generate energy while concurrently eliminating contaminants from wastewater via microbial metabolic processes, has emerged as a notable environmentally sustainable solution to meet the worldwide need for power generation. The microbial fuel cell (MFC) is a noteworthy technology among the options available for tackling the difficulties related to water and energy. It exhibits promise in simultaneously treating wastewater and producing power, as highlighted by Naha et al. 2023; Ishaq et al., 2023). A MFC is a bioelectrochemical device that can transform the chemical energy present in organic substrates into electrical power through direct means (Li et al. 2013). Electroactive microorganisms are employed for the dual purpose of waste treatment and energy generation (Sonawane et al. 2022). The purpose of this technique is accomplished through the stimulation of electric current by means of electron transfer. The transmission of electrons is aided by the process of microbial oxidation of reduced compounds, which serve as electron donors, occurring on the anode. Subsequently, the electrons are transported to oxidised molecules, such as oxygen, located on the cathode. The process of electron transfer is facilitated by the existence of an external electrical circuit (Naha et al. 2023). 

In comparison to anaerobic digestion, microbial fuel cells (MFCs) exhibit a high level of suitability for the remediation of soluble organic compounds with low concentrations, such as volatile fatty acids (VFAs). Additionally, MFCs demonstrate increased resilience to unfavourable environmental conditions, including low temperatures (≤ 20°C) and acidic pH levels. Moreover, the application of MFC technology can augment the retrieval of bioenergy in combination with anaerobic digestion, as demonstrated by Li et al. (2013). Various types of membranes, including the proton exchange membrane (PEM), anion exchange membrane (AEM), and cation exchange membrane (CEM), are utilised in the context of MFC technology. Prior research has shown evidence for the effectiveness of MFCs in the elimination of heavy metals (Wang et al. 2022; Aleid et al. 2023), nutrients (You et al. 2016; Ye et al. 2020), complex pharmaceutical compounds (Song et al. 2013; Wang et al. 2017), micropollutants (Wang et al. 2015), and several other contaminants from wastewater (Ahtesham et al. 2023).

Furthermore, the functioning of microbial fuel cells (MFCs) results in the generation of bioelectricity, presenting the possibility of economic advantages (Sibi et al. 2023). The microbial fuel cell (MFC) has developed as an ecologically conscious electrical technology and has undergone fast development as a sustainable system. This is mostly attributed to its distinctive capacity to address wastewater treatment and electricity generation concurrently. The operational mechanism of microbial fuel cells (MFCs) is predicated on the pivotal involvement of microorganisms in facilitating oxidation processes at the anode. These reactions are responsible for the degradation of organic matter and the generation of electrons and protons. In the anode chamber of an MFC, you typically find a substrate, which can be synthetic or real wastewater, along with a culture of microorganisms, either single or mixed. During this process, the protons are discharged into the anolyte and traverse the proton-selective membrane via diffusion, whereas the electrons are gathered by the anode electrode and conveyed through an external wire to the cathode, thereby generating electric current at the anode. At the cathode, the ultimate electron acceptor, commonly oxygen, undergoes reduction and gives rise to the formation of water (Boas et al. 2022).

MFCs can be classified into two categories based on their electron transfer pathways: mediator-type MFCs, in which microorganisms lack active surface proteins for direct electron transfer to the anode electrode, and mediator-less type MFCs. Furthermore, MFCs can be categorised by their design, encompassing double-chambered MFCs with separate anode and cathode chambers separated by an ion-exchange membrane, single-chambered MFCs that house both electrodes in a single chamber without physical separation, up-flow MFCs that enable vertical substrate flow, and triple-chambered MFCs featuring anode, cathode, and middle chambers separated by membranes, allowing for controlled conditions and component separation. These classifications help elucidate microbial fuel cells’ diverse configurations and functions in various applications (Naha et al. 2023). MFCs find application in multiple fields, including bioremediation, desalination, environmental monitoring, as well as hydrogen and chemical synthesis. These applications include energy generation and leachate treatment (Boas et al. 2022).

In a study conducted by Hassan et al. (2018), MFCs were investigated for their ability to utilise both young and old landfill leachate substrates to remove pollutants and generate renewable energy simultaneously. The study also delved into the analysis of the microbial communities at the anode. In batch mode, the MFC system achieved a power output of 96.8 mWm^−2^ and exhibited a chemical oxygen demand (COD) removal rate of 90.0 ± 1.2% when 60% young leachate was used as the substrate. However, when the system operated continuously with 100% young leachate, the power output decreased to 75 mWm^−2^, accompanied by a COD removal rate of 55.5%. Furthermore, the study demonstrated that power generation was feasible using simulated wastewater lacking an organic carbon source, indicating that ammonium could serve as a fuel source in the MFC. Interestingly, increasing the dosage of ammonium enhanced the overall system performance, but there was a point at which the inhibitory effect became more pronounced. The study also observed nitrogen removal, with 66.0 ± 3.3% removal of NH_4_^+^ − N and 86.0 ± 0.1% removal of NO_2_^−^ − N, following distinct removal pathways. In another study conducted by Mittal et al. (2023b), they examined how water loss due to evapotranspiration (ET) affected a CW-MFC using real municipal wastewater. Over 48 h, significant water loss (336 mm) occurred in the cathode zone due to ET. This improved cathodic reduction, increasing voltage from 182.5 ± 12.5 mV (first 6 h) to 800 ± 13.47 mV (48 h). Current and power density reached 85.71 mA/m^3^ and 25.71 mWm^−3^. Coulombic efficiency was 11.95%, and net energy recovery was 2.44 Wh/kg COD. Internal resistance decreased from 1000 to 700 Ω. After 48 h, the CW-MFC achieved high removal efficiencies: COD 80 ± 7.98%, ammonium 73.17 ± 5.01%, and phosphate 75.60 ± 1.65%. This study demonstrates ET’s significant impact on CW-MFC performance in real conditions. In a separate investigation conducted by Elmaadawy et al. (2020), an innovative cathodic algal biofilm microbial fuel cell (AB-OCU-MFC) was introduced as a means to enhance leachate treatment containing biorefractory organic compounds and high concentrations of ammonium nitrogen. The AB-OCU-MFC outperformed standalone algal biofilm-MFCs and control reactors in terms of COD, NH_4_^+^ − N, and TN removal, as well as algal biomass yield. Particularly, the AB-OCU-MFC equipped with a 2-cm-thick oxygen-consuming unit (OCU) achieved remarkable results, removing over 86% of COD, 89.4% of NH_4_^+^ − N, and 76.7% of TN. It also generated a peak voltage of 0.39 V and exhibited a biomass productivity of 1.23 g·L^−1^·d^−1^.

Furthermore, high-throughput sequencing of DNA revealed a significant shift in the microbial community within reactors that incorporated the OCU. Notably, the ratio of exoelectrogenic bacteria on the anode to denitrifying bacteria on the cathode substantially increased. These findings suggest that the cathodic algal biofilm MFC, incorporating a low-cost and bioactive OCU barrier, offers a promising approach for practical applications in MFC technology. The study presents valuable insights into the potential of AB-OCU-MFCs for efficient leachate treatment. It highlights the positive impact of the OCU on both microbial communities and system performance, offering new perspectives for real-world MFC applications.

While numerous studies have independently explored MFCs, FO processes, and the integration of membrane technologies with bioelectrochemical systems (BES), there still needs to be more research that combines these elements to tackle the pressing issues of water and energy scarcity. Specifically, prior work has yet to be found that comprehensively investigates the feasibility of using COD concentration regulation from leachate as a means of energy recovery while concurrently addressing the treatment of landfill leachate. This research gap is particularly significant given the increasing global concern over water contamination and the urgent need to develop sustainable approaches for treating landfill leachate, which contains a complex mixture of recalcitrant organic compounds. Moreover, the growing emphasis on renewable energy sources and the quest for energy-efficient wastewater treatment processes underscore the timeliness and relevance of this study. By addressing this gap, our research contributes to a more holistic and eco-friendly solution to the challenges posed by landfill leachate, offering a novel approach that integrates energy recovery and wastewater treatment.

While the fields of microbial fuel cells (MFCs), forward osmosis (FO) processes, and the integration of membrane technology with bioelectrochemical systems (BES) have been extensively explored independently, there exists a critical research gap in the comprehensive examination of their combined potential to address the interconnected challenges of water and energy scarcity. This study aims to fill this void by investigating the feasibility of utilizing chemical oxygen demand (COD) concentration regulation from leachate for energy recovery while concurrently addressing landfill leachate treatment. The urgency of our research is rooted in the global concern over water contamination and the imperative to develop sustainable approaches for treating landfill leachate, known for its complex mixture of recalcitrant organic compounds. Traditional methods often fall short, underscoring the need for innovative solutions.

The uniqueness of this research from other works is the integration of MFCs into landfill leachate treatment, a novel approach that concurrently addresses wastewater treatment and energy recovery. Previous work has not comprehensively explored this unique combination, making our study a pioneering effort in providing a holistic and eco-friendly solution to the challenges posed by landfill leachate. Considering the increasing emphasis on renewable energy sources and the demand for energy-efficient wastewater treatment processes, our research becomes timely and relevant. By addressing the research gap, we contribute to both environmental preservation and the evolution of sustainable waste treatment practices.

The primary objective of this research is to introduce the utilization of MFCs in treating landfill leachate, leveraging the innovative aspect of integrating advanced environmentally friendly technology with the pressing need for sustainable waste management strategies. This involves harnessing the distinct capabilities of MFCs to effectively treat landfill leachate, known for containing intricate and resistant organic contaminants. This study explores the hitherto unexplored realm of landfill leachate treatment through MFCs, aiming to introduce a novel and ecologically sustainable method. Our research seeks to revolutionize conventional leachate management by offering a unique, energy-positive, and resource-efficient alternative that not only treats leachate effectively but also contributes to renewable energy generation, marking a paradigm shift in sustainable waste treatment practices.

## Methodology

### MFC description and experimental setup of dual-chamber MFC

**Fig. 1:**
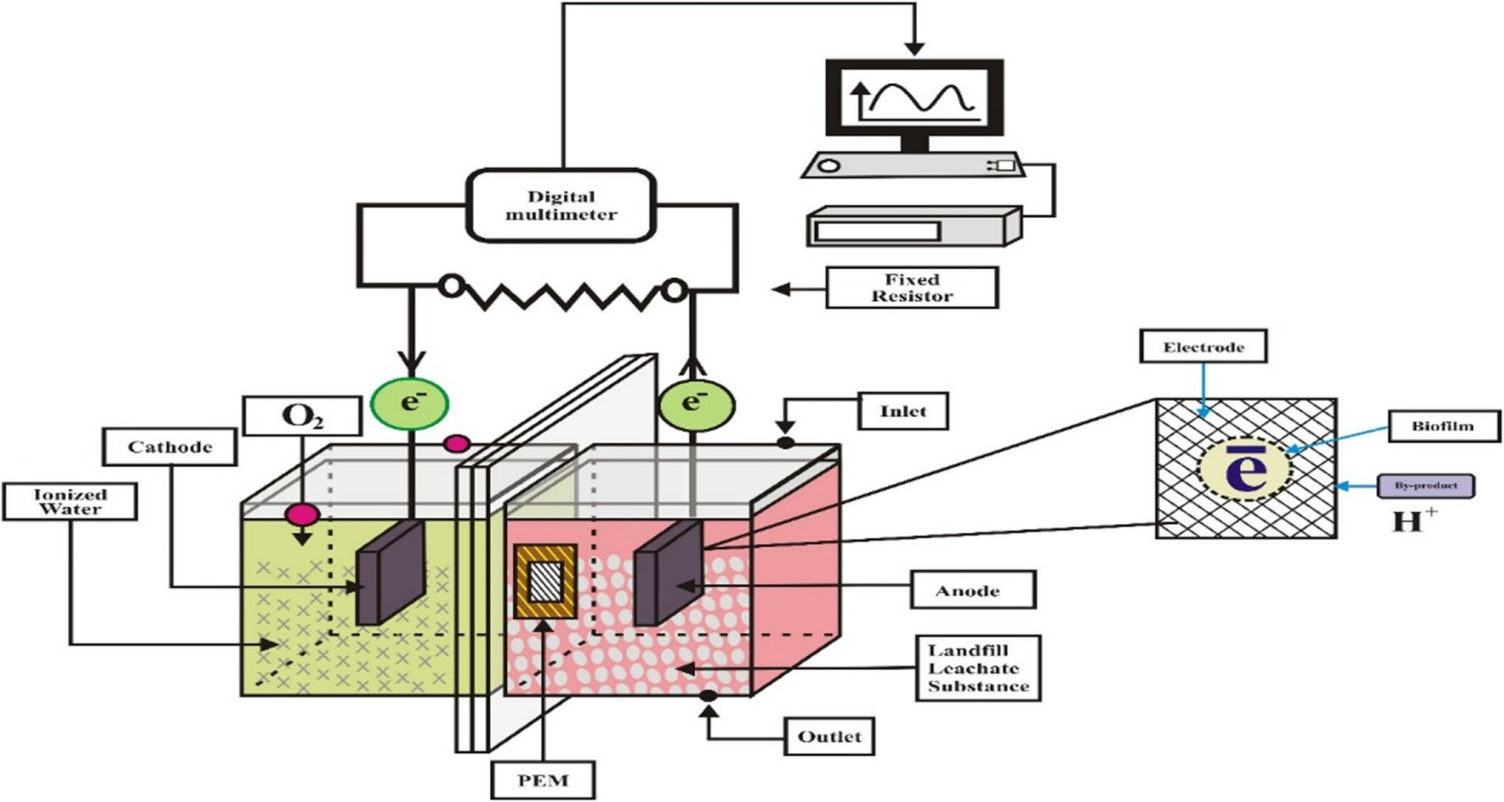
Schematic illustration of the experimental setup of the MFC

The studies were performed in a batch manner utilising a double-chamber microbial fuel cell reactor while maintaining a consistent temperature of 30°C and atmospheric pressure. The design of the microbial fuel cells (MFCs) was influenced by previous research, with some modifications implemented as outlined in the investigations conducted by Hassan et al. (2018) and Nor et al. (2015). The laboratory-scale reactors were constructed using transparent Plexiglas material and were assembled according to the schematic designs shown in Fig. 1. The microbial fuel cell (MFC) consisted of two compartments: a cathode compartment and an anode compartment. Each compartment had a capacity of 125 mL and a working volume of 100 mL. The dimensions of the compartments were determined to be 5 cm × 5 cm × 4 cm, yielding a specific area of 9.86 cm^2^. The anode and cathode were fabricated utilising carbon felt electrodes, characterised by their dimensions of height (h) = 1.7 cm, width (w) = 0.6 cm, and length (l) = 1.7 cm. The distance between the electrodes was around 4 cm, and a Nafion 117 proton exchange membrane (PEM) was utilised as the partition between them. The carbon felt underwent a variety of preparation procedures in the experimental technique done by Ni et al. (2020). Initially, the specimens were subjected to a 20-min immersion in acetone. Following that, the carbon felt underwent immersion in a solution containing 0.1 M hydrochloric acid (HCl) and was heated to boiling for 15 min. Subsequently, a thorough cleansing procedure was conducted with deionised water with the purpose of eradicating any contaminants that may have been present on the surfaces of the electrodes. The Nafion 117 membrane, manufactured by DuPont in Delaware, USA, underwent a pre-treatment process involving immersion in a solution of 0.1 M H_2_SO_4_, followed by 0.1 M H_2_O_2_ and subsequent rinsing with deionised water. Each stage in the process required 60 min while being conducted at a temperature of 60°C. The data were collected automatically through the utilisation of a digital data logger known as LABJACK HV. In order to link the electrodes, external resistances of 1 KΩ were employed, with 0.5-mm-diameter copper cables being utilised for this purpose.

### Biofilm development, anolyte and catholyte preparation

Complex microorganisms from the anaerobic sludge sourced from the anaerobic secondary digester at the Simpang Rengam wastewater treatment plant in Johor, Malaysia, were introduced into their respective microbial fuel cells (MFCs) to cultivate bacterial consortia, which acted as an inoculation source due to their suitable mixed bacterial composition. The MFCs were supplied with a medium enriched with peptone (0.5 g/L), yeast extract (0.5 g/L), and glucose (1 g/L) to support the growth of the consortia. The pH of the anolyte was maintained at 6.88, and the temperature was kept at 30 °C to create favourable conditions for anaerobic microorganisms. Similarly, the anodic chamber was purged with nitrogen gas before adding substrates and inoculants to eliminate oxygen and establish appropriate anaerobic conditions. The catholyte chamber contained distilled water and received a continuous supply of oxygen via an aquarium pump. This continuous aeration was performed at the top of the cathode chamber to ensure proper mixing and air bubbling. The pH of the anolyte chamber was adjusted to 6.88 using sodium hydroxide to maintain suitable conditions for anaerobic microorganism activity.

### Experimental process design

The efficiency of the batch system MFC was assessed by varying the influent chemical oxygen demand (COD) concentrations. Specifically, the MFC’s performance was examined under different COD levels: 1325 mg L^−1^, 1825 mg L^−1^, 2325 mg L^−1^, 2825 mg L^−1^, 3325 mg L^−1^, and 3825 mg L^−1^, all with a constant NH_4_-N concentration of 800 mg L^−1^. Voltage, current, and power density were calculated based on the substrate concentration and residence time, assessing their impact on removal efficiency. Growth kinetics were investigated to estimate the time needed for bacteria to reach a stable state. Optical density (OD) of bacterial growth at various organic matter concentrations was measured through periodic sampling every 4 to 6 h, with light absorption at 600 nm determined using a photometer (Model 6000, HACH). Additionally, the development of a biofilm layer on the anode was analysed using scanning electron microscopy.

### Data analysis

The stable voltage potential was recorded at 60-s intervals without an external resistor for the initial 21 days until a consistent voltage level was achieved. Subsequently, 1 KΩ resistors were connected via a LabJack U3-V Multimeter with built-in data logging capability (Agilent Technologies, California, USA), which was linked to a computer for recording voltage, current, and power. The current was computed using the formula I = V/R, and power density was calculated using P = IV, where P represents power in watts, V is voltage, I is current, and A signifies the electrode’s surface area. Current density was determined with the formula J = I/A, where J represents current density, I is current, and A is the surface area of the electrode. A consistent value of this parameter indicates the attainment of steady-state conditions. Analytical measurements, including current density and coulombic efficiency, were also conducted. The power density curve was employed to evaluate the electrical power generation potential. Polarisation data were obtained by disconnecting the MFC electrode (open circuit) for 2 h to maximise power output. The external circuit resistance was varied from 100 to 5000 Ω through a multimeter recorder (LABJACK U3-HV) connected to a personal computer for a maximum of 20 min for each resistance while monitoring the decreased voltage as the current increased. This experiment was concurrently monitored for identical sets of MFCs under continuous fed-batch operation mode. The polarisation curve was generated by plotting power density against current density. The Coulombic efficiency, CE (%), which represents the recovery of electrons, was calculated using Eq. 1 adopted from (Suransh et al. 2023).

CE serves as a measure to assess how many electrons are produced through the oxidation of organic matter compared to the theoretically expected amount. Alternatively, CE can be expressed as the fraction of organic matter that is utilised for energy generation. It is worth noting that some non-electroactive bacteria may utilise organic matter for their growth and carbon storage purposes, whereas electrogenic bacteria employ it for metabolic functions. Consequently, a low CE may suggest the presence of only a limited number of electrogenic bacteria within the system (Kaixuan et al. 2020; Suransh et al. 2023).

$${\text{CE}}=\frac{8\underset{0}{\overset{t}{\int }}1 {\text{dt}}}{ F.\mathrm{\Delta COD}.V{\text{An}}} \times 100\%$$

In the formula for CE, CE represents the CE in percentage (%), “t” denotes time in seconds (s), “I” represents the achieved current of the MFC system in amperes (A), “F” is Faraday’s constant, which has a value of 96,485 coulombs per mole (C/mol), “VAn” signifies the volume of the anolyte for batch operation in litres (L), ΔCOD represents the changes in organic matter removal in milligrams per litre (mg/L).

### Polarisation curve

The voltage of an MFC system is represented by a curve known as the polarisation curve, which is a function of the current density. This curve can be divided into three interconnected regions: activation loss, ohmic loss, and mass transport loss. The power density curve is utilised to assess the system’s capacity for generating electrical power. Polarisation data were collected by disconnecting the MFC electrode (open circuit) for 2 h to maximise power output. The external circuit resistance was varied between 100 and 5000 ohms using a multimeter recorder (LABJACK U3-HV) connected to a computer, with each resistance setting observed for a maximum of 20 min while measuring the reduced voltage as the current increased. This experiment was simultaneously conducted for identical sets of MFCs under continuous fed-batch operation mode.

### Statistical analysis using multiple correspondence analyses

A multivariate statistical approach that works well with categorical data is the MCA. A multiple correspondence analysis (MCA) is used to examine the removal efficiency for the treatment in an objective manner, the potential links among all the variables, and to find particular profiles (removal efficiency among the pollutant class). Multivariate correspondence analysis (MCA) is a statistical technique used for the purpose of exploring the relationship between many variables within a category dataset. The MCA software tool facilitates the transformation of tabular data into visual representations, enabling users to see and comprehend the information contained within the data effectively. According to Chauvin et al. (2013), a substantial number of variables may be represented as points in low-dimensional Euclidean spaces. Graphical depictions of relationships among variables are employed. The graphics aim to portray both the similarities and differences across the profiles by emphasising the dimensions that explain the majority of the variability in the data.

Consequently, the pertinent variables in this investigation have been reduced to three dimensions, which explain the majority of the variation seen in the experimental dataset. The dataset is represented as vectors when projected into a three-dimensional space. Without making any presumptions about the inherent organisation of the data, the arrangement of the variable categories inside this three-dimensional framework signifies their interdependent connections. The classification points inside a graph can be represented and formed utilising the percentage coordinates along the *x*- and *y*-axes. From a statistical perspective, it may be inferred that categories that exhibit proximity in a plot are likely to have a significant relationship. If two categories exhibit high coordinates and spatial proximity, they probably share a close relationship. When two categories have high coordinates but are spatially far from one other, it suggests the presence of an inverse link between them. The concept of inertia is associated with the explained variance of the dimensions. While maintaining the integrity of category connections, the interpretation of findings can be enhanced by utilising eigenvalues and factor scores. This analysis is employed to answer the following questions;

i. Are there gaps in the removal efficiency that could be filled by employing new treatment?
ii. Is the removal efficiency adequate in treating the pollutant, correct?
iii. Could the treatment differentiate itself from the pollutant type and their concentration?

## Results and discussion

The fed-batch operation mode is widely recognised as crucial for optimising the conditions that yield maximum electrical output in MFCs. Polarisation curves were generated to evaluate the MFC reactor’s performance, and peak power output was determined. These assessments were conducted at room temperature, maintaining a pH range of 6.88 to 7.0. The results provided evidence of the presence of the necessary microorganisms in landfill leachate. The outcomes of the biofilm development experiments are presented in Table 1.

**Table 1:** Characteristics of landfill leachate used in this study

Parameter	Unit	Concentration
BOD	mg L^−1^	112 ± 7
COD	mg L^−1^	1322.5 ± 5
BOD_5_/COD	-	0.0845 ± 1.4
NH_4_-N	mg L^−1^	411 ± 4
Turbidity	NTU	134.95 ± 8
pH	-	6.88 ± 3
Temperature	^0^c	30 ± 2.5
Conductivity	μs.cm	107746 ± 7.4
NO_3_-N	mg L^−1^	70 ± 5.5
NO_2_-N	mg L^−1^	110 ± 8.5

Table 1 presents the properties of the landfill leachate used in this study, which are essential water quality parameters crucial for assessing environmental conditions. Notably, the data reveals concentrations of BOD (112 ± 7 mg L^−1^) and COD (1322.5 ± 5 mg L^−1^), indicating significant water organic and chemical pollutant loads. According to Bader et al. (2022), if the BOD:COD ratio is greater than 0.6, the waste is considered biodegradable and can be effectively treated biologically; if the ratio is between 0.3 and 0.6, seeding is necessary to treat it biologically due to slow degradation process; if the ratio is less than 0.3, biodegradation is not possible and the wastewater inhibits bacterial seed activity. The low BOD_5_/COD ratio (0.0845 ± 1.4) suggests limited biodegradability of organic matter. A leachate with a low BOD to COD ratio reveals a considerable abundance of non-biodegradable organic contaminants, suggesting that the leachate is less inclined towards embracing natural biological treatment procedures. Ammonium nitrogen (NH_4_-N) registers at 411 ± 6 mg L^−1^, while turbidity stands at 134.95 ± 8 NTU, indicating the presence of suspended particles. High ammonia nitrogen concentration signifies that the leachate is highly polluting and requires treatment before it can be safely discharged into the environment. Elevated turbidity can hinder light penetration, affecting photosynthesis and disrupting aquatic life habitats. Investigating the source of these particles is crucial for assessing potential pollution origins. The slightly acidic pH level is 6.88 ± 0.3, and high conductivity (107,746 ± 7.4 μs/cm) suggests significant dissolved ion presence, potentially stemming from industrial or geological factors. Nitrate nitrogen (NO_3_-N) is at 70 ± 5.5 mg L^−1^, and nitrite nitrogen (NO_2_ − N) measures 110 ± 8.5 mg L^−1^, contributing to the assessment of inorganic nitrogen levels. These nitrogen species can originate from various sources, impacting water quality and ecosystems. High concentrations of nitrate and nitrite in leachate from landfills are significant because they pose serious health risks to both humans and ecosystems. Nitrate and nitrite can lead to methemoglobinemia (blue baby syndrome) in infants and contribute to the pollution of water sources, affecting aquatic life and overall water quality. The 30 ± 2.5°C temperature is vital for aquatic ecosystems, influencing organism metabolism, gas solubility, and biological processes. Monitoring temperature aids in assessing ecosystem health and identifying potential thermal pollution.

### Power production and removal efficiencies at variable COD loading rate

**Fig. 2:**
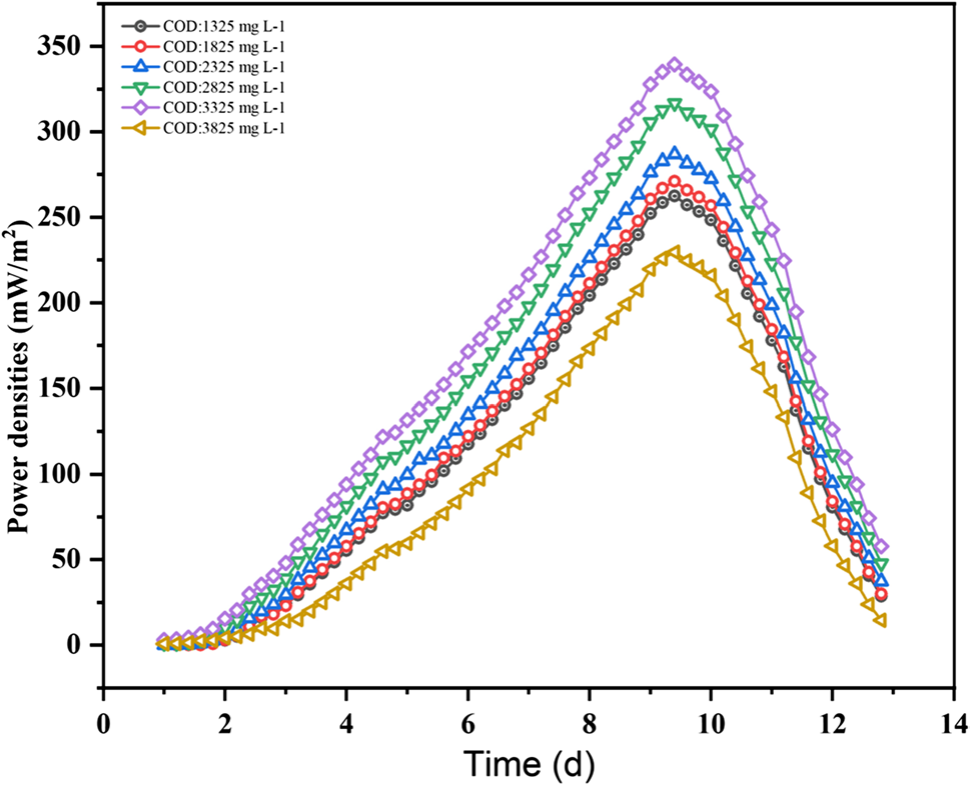
Plots of power densities at different COD concentrations

The COD concentration in the feed to the anode chamber ranged from 1325 to 3825 mg L^−1^, while NH_4_-N was maintained at 800 mg L^−1^, and the residence time was consistent in the anode chamber. The pH of the anode solution was controlled at 6.88 to 7.0 using a 0.1 M phosphate buffer. The experiment was conducted at room temperature, and distilled water, aerated for oxygen supplementation, served as the cathodic electron acceptor. Various parameters, including voltage, current, and substrate removal, were continuously monitored. Notably, within the studied range of COD concentrations, the MFC exhibited a linear increase in current output as COD removal from the leachate substrate increased. The impact of varying COD loading rates on power generation and treatment efficiency is intricate and is further elucidated in Fig. 2.

The impact of varying COD loading rates on power generation and treatment efficiency is indeed complex, and this study aimed to investigate the experimental performance of different COD concentrations in MFCs. The specific COD concentrations and their corresponding power densities (in parentheses) are as follows: 1325 mg L^−1^ (262.34 mWm^−2^), 1825 mg L^−1^ (271.01 mWm^−2^), 2325 mg L^−1^ (286.96 mWm − ^2^), 2825 mg L^−1^ (316.75 mWm − ^2^), 3325 mg L^−1^ (339.41 mWm^−2^), and 3825 mg L^−1^ (229.52 mWm^−2^).

**Fig. 3:**
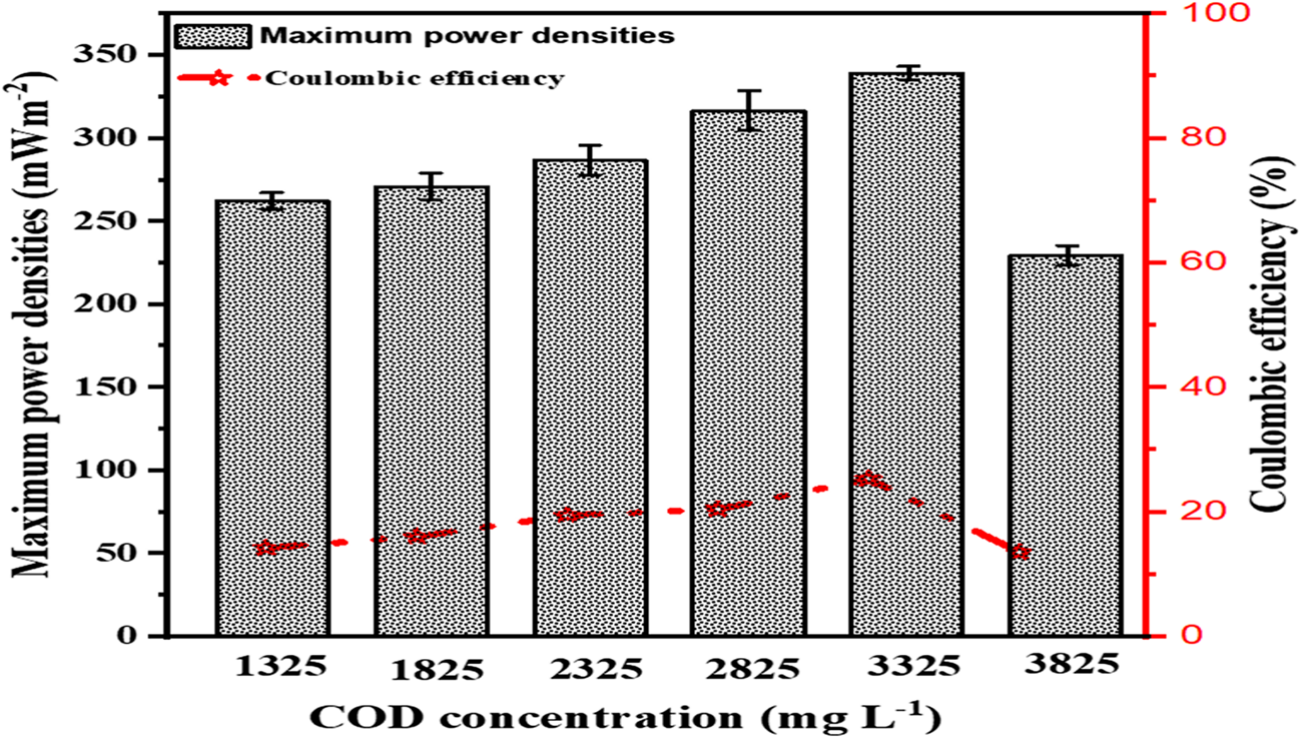
Plots of maximum power densities and coulombic efficiencies at variable COD concentrations

Figure 3 shows a linear relationship between power density and COD concentration. As the COD concentration increases from 1325 to 3325 mg L^−1^, there is an overall increase in power density. This indicates that an increase in the organic matter content of the MFC system leads to enhanced microbial metabolism and electron transfer activities, which in turn leads to greater power generation. The power density at the maximum COD concentration of 3825 mg L^−1^ was 229.52 mWm^−2^, lower than the peak power density of 339.41 mWm^−2^. Since the highest power density is recorded at a COD concentration of 3325 mg L^−1^, it can be concluded that this value is where the MFC produces the most efficient amount of energy. The decline in power density beyond the COD concentration of 3325 mg L^−1^ could be attributed to the high COD and low BOD:COD ratio which indicates high non-biodegradable organic matter and other chemicals content of leachate. Another reason for this decline could be attributed to the increase in toxicity of materials as a result of the increase in COD concentration. It has been shown that when COD concentrations increase, the concentration of toxic materials increases and also flocculation of chemicals may occur (Song et al. 2013; Musa and Ahmad 2010). The reduction in power density at higher COD concentrations emphasises the importance of maintaining a balanced organic matter content and C/N ratio to ensure efficient power generation and treatment within the MFC system. These results underscore the significance of COD concentration as a critical factor for electricity generation in MFCs.

The potential for energy production in an MFC is directly proportional to the concentration of organic matter, specifically chemical oxygen demand (COD), in the substrate. Higher COD content provides the microorganisms in the MFC with more organic materials to oxidise, increasing their ability to generate electrical currents. The Coulombic efficiency (CE) of the substrate reflects how effectively it converts chemical energy into electrical current. As illustrated in Fig. 3, this research focused on both parameters to evaluate and enhance MFC performance in leachate treatment and bioenergy generation. Additionally, researchers have explored the current carrying capacity of biomass materials and the feasibility of recovering the energy produced within the MFC system (Hu et al. 2017). Recoverable energy can range from 2.0 to 50.0% in MFC systems, particularly for easily biodegradable materials like glucose, amino compounds, and organic acids (Pandey et al. 2016). The electrons generated in an MFC reactor have various potential applications, including electricity production and bio-electrochemical processes (Choudhury et al. 2021).

Figure 3 reveals that the highest CE (25.5%) was achieved at a COD concentration of 3325.0 mg L^−1^, and it decreased to 13.56% at 3825.0 mg L^−1^ of COD. This decline in CE may be attributed to the production of intermediate metabolites, such as acetate, butyrate, and propionate, during the breakdown of carbohydrates by microbes, which can adversely affect CE (Rahimnejad et al. 2014). Previous studies (Rahimnejad et al. 2014) have demonstrated that MFC systems fed with simple substrates like glucose or acetate generally yield higher CEs compared to those fed with actual wastewater. However, a CE of 13% was considered quite low in these studies. Similar results were reported for xylose in both fed − batch and continuous processes (Rahmani et al. 2022), where microbial metabolism of sugar led to the release of intermediates that lowered CE (Rahimnejad et al. 2015).

The intricate interplay of parameters affecting MFC operation explains why high CE and low COD concentrations, as well as low CE with high COD concentrations, can coexist. The presence of dissolved ions, possibly from ionic species or salts in the influent wastewater, can increase electrical conductivity without significantly affecting organic matter content. This is why high CE may sometimes be observed alongside low COD content. Conversely, water with low CE and high COD concentration likely contains numerous easily oxidisable organic compounds but few dissolved ions that impact conductivity (Priya and Hait 2018). Consequently, the composition of wastewater and its contaminants can lead to a wide range of CE and COD values in wastewater samples.

**Fig. 4:**
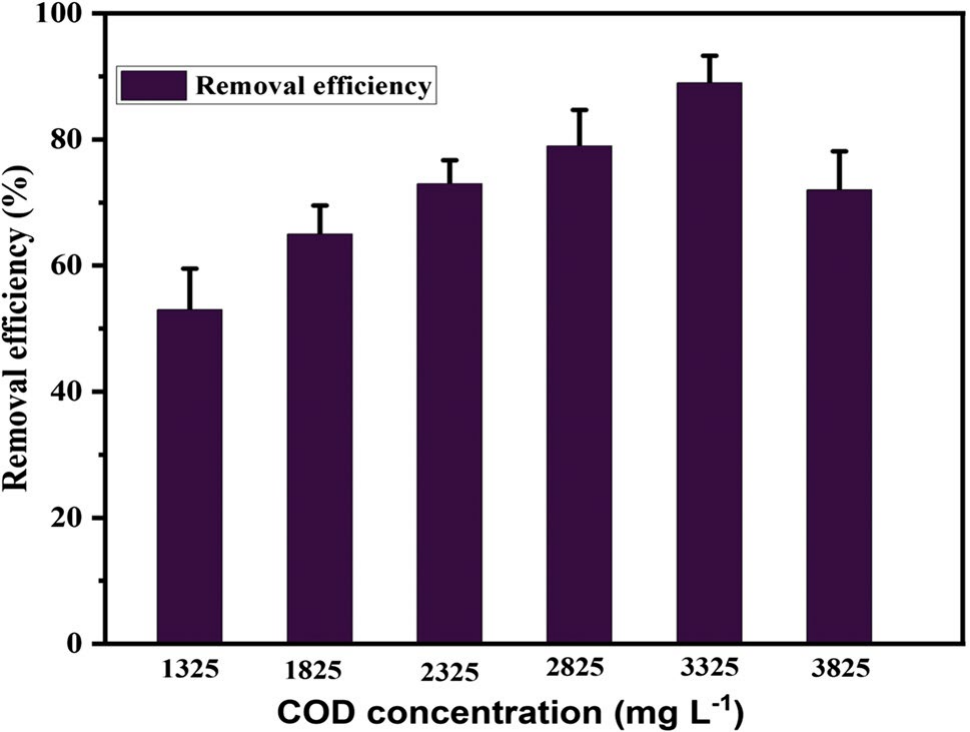
COD removal efficiency (%) in MFC with different initial COD concentrations

The evaluation of the performance of the linked microbial fuel cells (MFCs) system, which employed leachate as a substrate with different starting chemical oxygen demand (COD) concentrations, was conducted by analysing the COD removal efficiency (refer to Fig. 4). The inclusion of carbon content within leachate wastewater functions as a source of electron donors in metabolic processes, resulting in the concurrent decomposition of the substrate and the generation of electricity. At a relatively low concentration of COD of 1325 mg L^−1^, there was a buildup of organic acids as a consequence of biodegradation occurring in the anode compartment. The observed phenomenon suggests a significant presence of organic materials, which subsequently results in an elevated level of anodic acidification. As a result, the prevailing circumstances were unfavourable for the activity of microorganisms, leading to a decrease in the effectiveness of COD removal.

The highest COD removal percentage (89.0 ± 4.3%) was achieved at an initial COD concentration of 3325 mg L^−1^, NH_4_-N = 800 mg L^−1^, pH = 6.88, and ambient temperature in the anodic section. In contrast, the removal efficiencies for COD concentrations of 1325 mg L^−1^, 1825 mg L^−1^, 2325 mg L^−1^, 2825 mg L^−1^, and 3825 mg L^−1^ were 53 ± 6.5%, 65 ± 4.5%, 73.0 ± 3.7%, 79.0 ± 5.7%, and 72.0 ± 6.1%, respectively. This shows that the COD removal percentage consistently increases as the initial concentration of COD increases until it peaks at a COD concentration of 3325 mg L^−1^. It started showing a slight decrease as the initial COD concentration increased to 3825 mg L^−1^. While some studies have shown a consistent increase percentage of COD removal, e.g., from 61.1 to 80.5% (Kim et al. 2019), and 81 to 85% (Zhou et al. 2021); in other studies, the COD removal percentage in MFC does not consistently increase. In one study, the removal of TAN in MFCs increased the COD/TAN ratio, leading to further degradation of COD (Kim et al. 2019). However, in another study, while using MFCs increased the COD removal rate due to the current generation, secondary processes were needed to reduce COD to suitable levels for discharge (Zhang et al. 2015). In a microbial fuel cell designed for nitrate removal, the amount of COD decreased over time, but the COD removal percentage was not consistently reported (Ali and Maira Anam, Sameen Yousaf 2017). Finally, in a study on electrotrophic denitrification, the COD/SO4^2−^ ratio affected the performance of the process, with higher ratios enhancing electrotrophic denitrification but deteriorating sulfate reduction (Tao et al. 2022). The anodic chamber of the MFC functions as an anaerobic growth reactor, highlighting the high potential of MFCs as a replacement for biological treatment processes (Dong et al. 2014). The results indicate that using sludge and wastewater as the microbial source and fuel, respectively, yields the highest efficiency, eliminating the need for an additional microbial source, aligning with the findings of (Bajithun and Ethiraj 2022).

### Polarisation curves at different initial COD concentrations

**Fig. 5:**
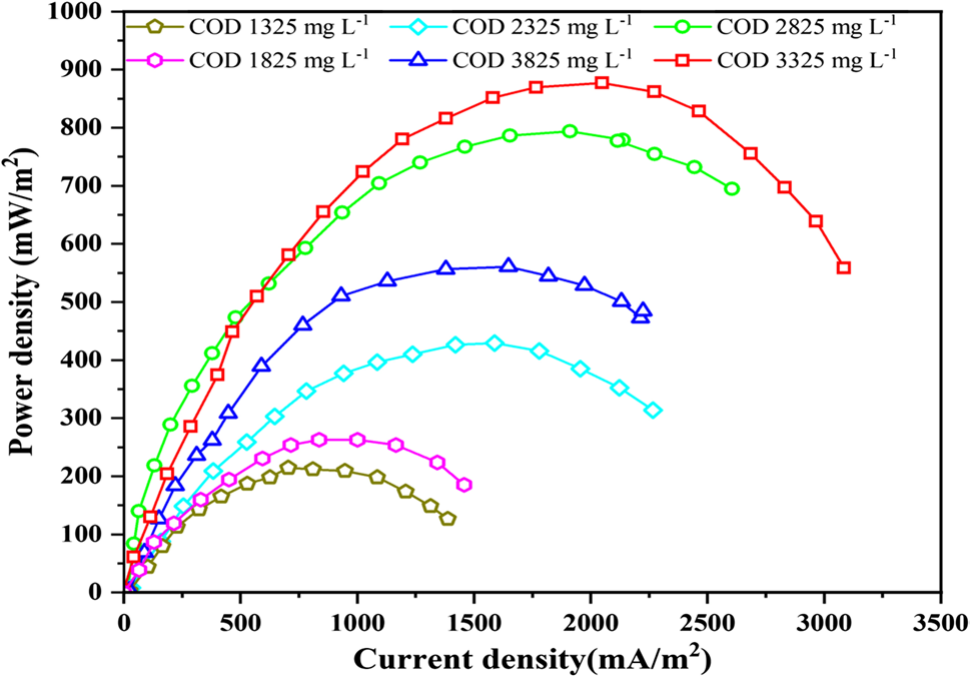
Power densities at different COD loading rates

The efficiency of the MFC reactor was assessed by monitoring voltage and calculating the maximum power density generation. To evaluate performance, the external resistance was adjusted after 20 min for each of the MFC reactors. Additionally, the anode surface area was normalised, and a polarisation curve was generated, as depicted in Fig. 5. This curve illustrates the relationship between power density and current density at various COD concentrations.

During polarisation, the highest recorded maximum power density was achieved in the MFC containing COD at 3325 mg/L, reaching 877.2 mWm^−2^. This was followed by the MFC with COD at 2825 mg/L (793.84 mWm^−^2), the MFC with COD at 3825 mg/L (560.84 mWm^−^2), the MFC with COD at 2325 mg/L (429.36 mWm^−^2), the MFC with COD at 1825 mg/L (262.69 mWm^−^2), and finally, the MFC with COD at 1325 mg/L (214.68 mWm^−^2), as shown in Fig. 5. These results suggest that air exposure and ultrasonication treatment enhanced the activity of the electrogenic biofilm grown on the anodes of the MFCs, resulting in lower methanogenic electron loss and increased power density. This finding aligns with previous studies (Rahmani et al. 2022). Previous studies have also shown that the highest recorded maximum power density in the MFC was achieved as the influent COD increased. In one study, the power density of the stacked MFC increased from 25.6 ± 2.5 to 42.1 ± 1.2 Wm^−3^ as the influent COD concentrations increased from 200 to 800 mg/L (Wu et al. 2022). Another study found that the MFC with a composite electrode achieved the highest power density of 66.9 ± 1.6 Wm^−3^, which was about 5.3 and 1.2 times higher than MFCs with graphite granules and a graphite fibre brush, respectively (Novitasari et al. 2022). These results indicate that raising the influent COD concentration can increase the power density in MFCs. According to the results of the current investigation, higher COD concentrations result in greater electron flow and higher power density in the MFC system because there are more organic substrates for microorganisms to digest.

Table 2 presents a comprehensive evaluation of the performance of microbial fuel cells (MFCs) under varying starting chemical oxygen demand (COD) concentrations. The data presented demonstrates a conspicuous pattern: when the initial concentration of chemical oxygen demand (COD) rises from 1325 to 3825 mg L^−1^, there is a notable increase in voltage, current density, and power density. The power density shown by the microbial fuel cells (MFCs) exhibits a consistent growth from 214.68 to 877.20 mWm^−2^, suggesting a positive correlation between electrical potential and greater COD concentrations. The observed rise in power density is concurrent with a proportional rise in current density, which exhibits an increase from 1387.09 to 3084.89 mA/m^2^. This augmentation signifies an accelerated rate of electron transfer and, subsequently, a heightened level of power generation (Rahmani et al. 2022).

**Table 2:** MFCs performance with different initial COD concentrations in power generation, current density, Columbic efficiency

S/N	COD conc. (mg L^−1^)	Maximum current densities (mAm^−2^)	Power densities (mWm^−2^)
1	3325	3084.89	877.20
2	2825	2605.36	793.84
3	3825	2223.84	560.84
4	2325	2265.95	429.36
5	1825	1457.14	262.69
6	1325	1387.09	214.68

Moreover, there is a positive link between Coulombic efficiency and COD concentration. This observation indicates that an increase in the initial concentration of COD in the leachate leads to enhanced efficiency of MFCs in the conversion of organic matter into electrical energy. It is important to note that the microbial community structure varies with different concentrations, and dominant bacteria can adapt successfully to higher concentrations (Wang et al. 2020). However, an increase in the initial voltage of MFC systems can be caused by a different source of organic matter (Reza et al. 2019). There is a slight decrease in power density at COD 3825 mg L^−1^, possibly due to the impact of microbial community structure influenced by concentration, which can compromise MFC performance.

Moreover, if COD exceeds a certain concentration, electricity production could be hindered due to substrate saturation (Santos et al. 2017), in line with previous findings by Wang et al. (2020). Notably, increasing COD in the anodic section can have a positive effect on power generation. In other words, implementing the system with higher COD and extended time leads to relatively good bioelectricity production (Kalathil et al. 2018). Furthermore, substrate quality (including porosity, material, and roughness) and the bacterial community that develops during adsorption can impact microbial activity and the maximum power generated from the leachate (Rahmani et al. 2022). However, dominant bacteria in the anode chamber tend to have stronger environmental adaptability under lower concentrations (Wang et al. 2020). Table 3 provides a comparative overview of various microbial fuel cell (MFCs) studies and their performance in terms of maximum power density and COD removal efficiency. The present study, which focuses on landfill leachate treatment using a dual-compartment MFC, is compared with other studies regarding feedstock, MFC type, maximum power density, and highest COD removal efficiency. There are numerous advantages of the present study over others. The present study deals with landfill leachate with a COD concentration of 3.32 g/L. This high COD concentration is a distinctive feature, as it represents a more challenging and complex wastewater compared to some other studies that dealt with lower COD concentrations or specific types of wastewaters (e.g., petrochemical, saline, municipal, aquaculture, hospital, dairy effluent). The present study demonstrates a maximum power density of 339.41 mW/m^2^. While this value is not the highest in the table, it is competitive and falls within the range observed in other studies. The notable point here is that despite the challenging feedstock of landfill leachate, the MFC in the present study performs well in terms of power generation. The present study achieves a COD removal efficiency of 89%. This is comparable to other studies in the table and is noteworthy considering the complexity of landfill leachate. The high COD removal efficiency indicates the effectiveness of the MFC in treating the wastewater. In summary, the present study stands out for its focus on landfill leachate, high COD concentration, and competitive performance in terms of power density and COD removal efficiency when compared to a diverse set of wastewater types and MFC configurations in the table.

**Table 3:** Performance comparison of the present study with related studies

S/N	Feedstock and COD concentration	MFC Type	Maximum power density (mWm^−2^)	Highest COD removal efficiency (%)	References
1	Petrochemical industrial wastewater of 1.5g COD/L	Dual-chambered microbial fuel cell	270	88	(Tamilarasan et al. 2024)
2	Wastewater	Coupled nitrification denitrification microbial fuel cell	1.27 W m^−3^	98	(Nguyen and Babel 2024)
3	Saline wastewater 2.9 g COD/L	Dual-chambered microbial fuel cell	162.09	76	(Vijay et al. 2023)
4	Municipal wastewater	Constructed wetlands integrated with microbial fuel cells (CW-MFC)	25.71	80	(Mittal et al. 2023a)
5	Aquaculture wastewater (1.25 g COD/L)	Saline anode MFC	369	91	(Pugazhendi et al. 2021)
6	Hospital wastewater	Composite constructed wetland and microbial fuel cell system	21.26 and 42.93	95.3	(Jain et al. 2023)
7	Dairy effluent treatment	Double chamber MFC having graphite electrodes and an anion exchange membrane	36.39	81	(Tabish et al. 2023)
8	Artificial saline wastewater (1.0–1.1 g COD/L)	Air cathode MFC	273	93	(Xin et al. 2022)
9	Seafood wastewater (1.25 g COD/L)	Air cathode MFC	530	89	(Pugazhendi et al. 2020)
10	Landfill leachate 3.32 g COD/L	Dual compartment MFC	339.41	89	Present study

### Effect of anodic pH on the performance and internal resistance

The microbial fuel cells (MFCs) were operated within the anode chamber, employing a hydraulic retention time (HRT) of 38 days. The pH of the leachate was precisely regulated between 6.88 and 7.0 by the application of a 50-mM phosphate buffer. The experiment involved maintaining each pH level for 12 days. The experiment was carried out under ambient room temperature conditions, which varied between 22 and 35 °C. Throughout the experiment, the cathodic electrolyte was comprised only of distilled water, which was subjected to aeration to enhance the cathodic reaction. The maximum recorded current was attained by maintaining the influent pH at a value of 6.88.

In contrast, a reduction in current was seen when the pH of the influent in the anode chamber exceeded 7.0 or dropped below 6.0. The results of this study indicate that microbial activity has a reduced rate at pH levels below the optimum value of 6.88. The pH of the electrolyte significantly influences the power generation in MFCs. Typically, MFCs demonstrate elevated levels of current production while operating at neutral pH levels (pH 7–8) in comparison to lower or higher pH levels (Asghar et al. 2017). According to Jalilluddin et al. (2015), the anodic microbial process in MFCs has a preference for a neutral pH. It has been shown that microbial activity tends to decrease at pH values that are either higher or lower than neutral.

Conversely, Sawasdee and Pisutpaisal (2016) found that the cathodic reaction in MFCs exhibits enhanced performance as the pH increases, resulting in a corresponding rise in power generation. The Coulombic efficiency displayed a comparable pattern but with more pronounced disparities than the current. The observed phenomenon can be attributed to a reduced rate of proton transfer resulting from a reduction in the concentration gradient of protons across the membrane. Furthermore, the pH value of 6.88 exhibits a preference for methanogenesis, hence leading to an increased elimination of COD. It is noteworthy that the internal resistance of the MFC exhibited a reduction when the pH difference between the anode and cathode increased. Upon a small rise in pH, the cell’s internal resistance was measured to be 213 ohms. An increased disparity in pH levels amplifies the pace at which protons traverse the proton exchange membrane (PEM), resulting in heightened power generation within the MFC. This phenomenon is consistent with the findings of a prior study conducted by Jadhav and Ghangrekar (2015).

### Determination of bacterial growth using optical density 600 nm

The optical density (OD) curve depicting variations in OD values at different COD loading rates offers insights into the responsiveness of the microbial population to changes in substrate availability. An increase in OD value with higher COD loading rates suggests that microorganisms can efficiently metabolise the substrate, potentially resulting in higher microbial activity, increased current generation, and greater power output from the MFC (Domańska et al. 2019).

**Fig. 6:**
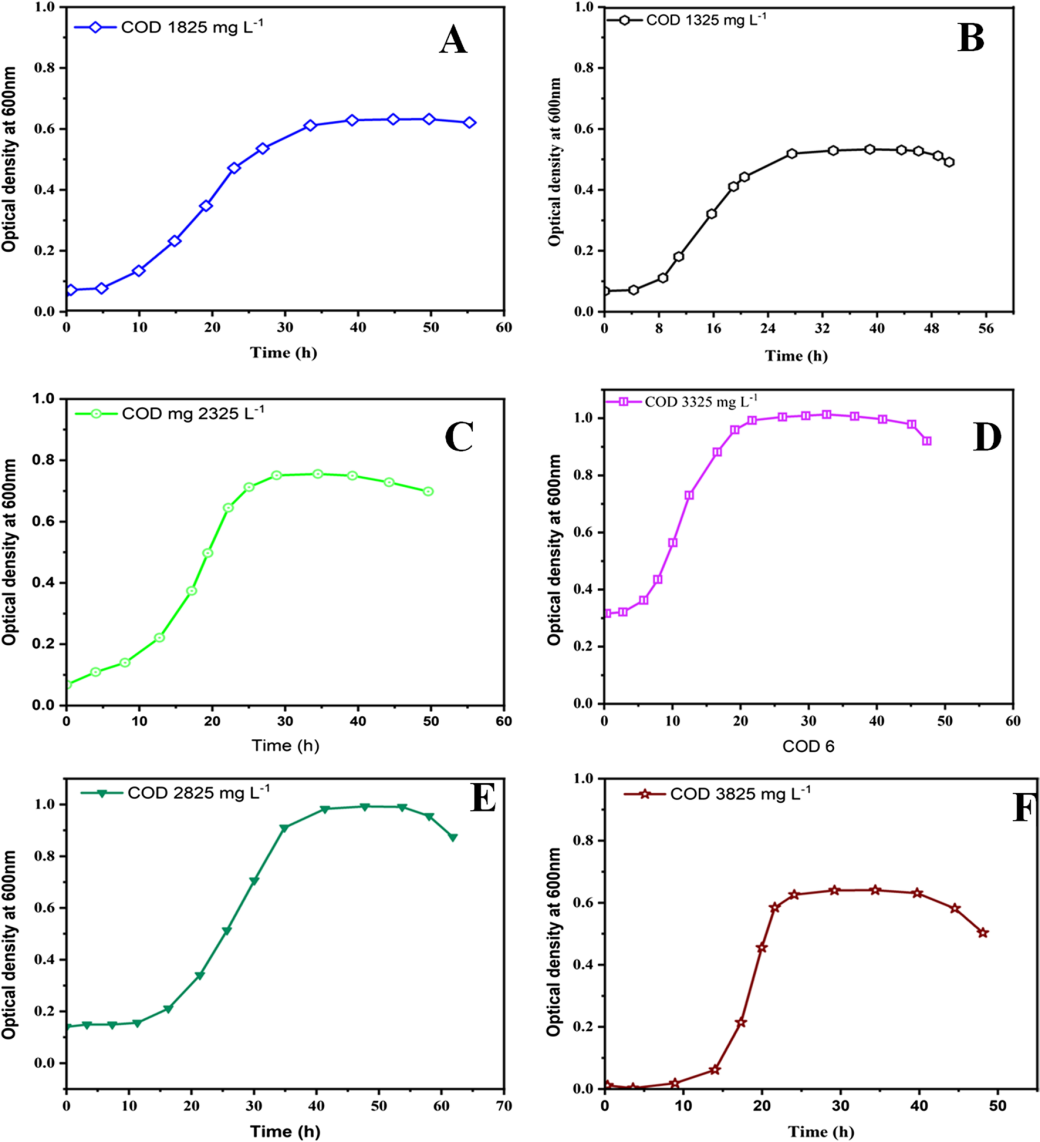
Bacterial growth curve (OD) as a function of time in different anodic chamber MFCs at different initial COD concentrations

Figure 6A, B, C, D, E, F reveals a typical growth pattern for microbial populations. It begins with an initial lag phase, where bacterial growth remains relatively constant at around 0.067. During this phase, microorganisms are adapting to their environment and may not be actively dividing. This period allows for the adaptation of microorganisms to new environmental conditions through the necessary enzymatic processes (Domańska et al. 2019). The low OD value indicates a state of low cell density. As time progresses, there is a microbial bloom, leading to a significant increase in OD, closely correlated with the degradation of organic materials. After approximately 30 h (for most cases), the growth rate stabilises, and the microbial culture enters a phase of steady growth for over 45 h. The growth curve indicates an increase in OD values from 0.01 to 0.90. Beyond the 15.7-h mark, the growth rate appears to slow down, signalling a transition from the exponential growth phase to the stationary phase. During this transition, nutrient limitations, the accumulation of waste products, and reduced cell division can occur. It is worth noting that the bacterial growth curve at various substrate concentrations showed a similar pattern, with a lag phase followed by a rapid transition into the logarithmic growth phase as microorganisms started multiplying. After 40–50 h, the bacterial growth rate stabilised, approaching the bacterial death rate. The stationary phase, which may result from nutrient depletion or other limiting factors, follows this phase. The stationary phase is essential for various microbial processes and can be associated with the production of secondary metabolites or the maintenance of long-term cultures. While the OD values continued to rise beyond this point, the growth curve entered the logarithmic death phase due to factors like time, nutrient depletion, and unfavourable conditions. Importantly, this phase reversal did not occur initially; instead, it was a consequence of changing conditions.

Figure 6A, B, C, D, E, F also shows that the highest value of power density obtained was at COD concentration of 3325 mg L^−1^ (Fig. 6D). The highest microbial growth, indicated by the maximum increase in OD value (1.02), was observed when the COD concentration was at 3325 mg L^−1^. This high growth is attributed to the abundance of nutrients available at this concentration. The microbial population remained in a constant growth stage for some time but eventually decreased sharply, likely due to an increase in the acidity of the medium. These findings contrast with those of Rahmani et al. (2022), who used glucose at variable COD concentrations and achieved a maximum cell density with an OD value of 1.4 at O.D 620 nm. This inconsistency may be due to differences in the type of substrate used (glucose vs. landfill leachate), which can significantly impact microbial growth dynamics and OD values. Additionally, other factors, such as the composition of the microbial community, environmental conditions, and the presence of inhibitory compounds, can also influence microbial growth patterns.

**Fig. 7:**
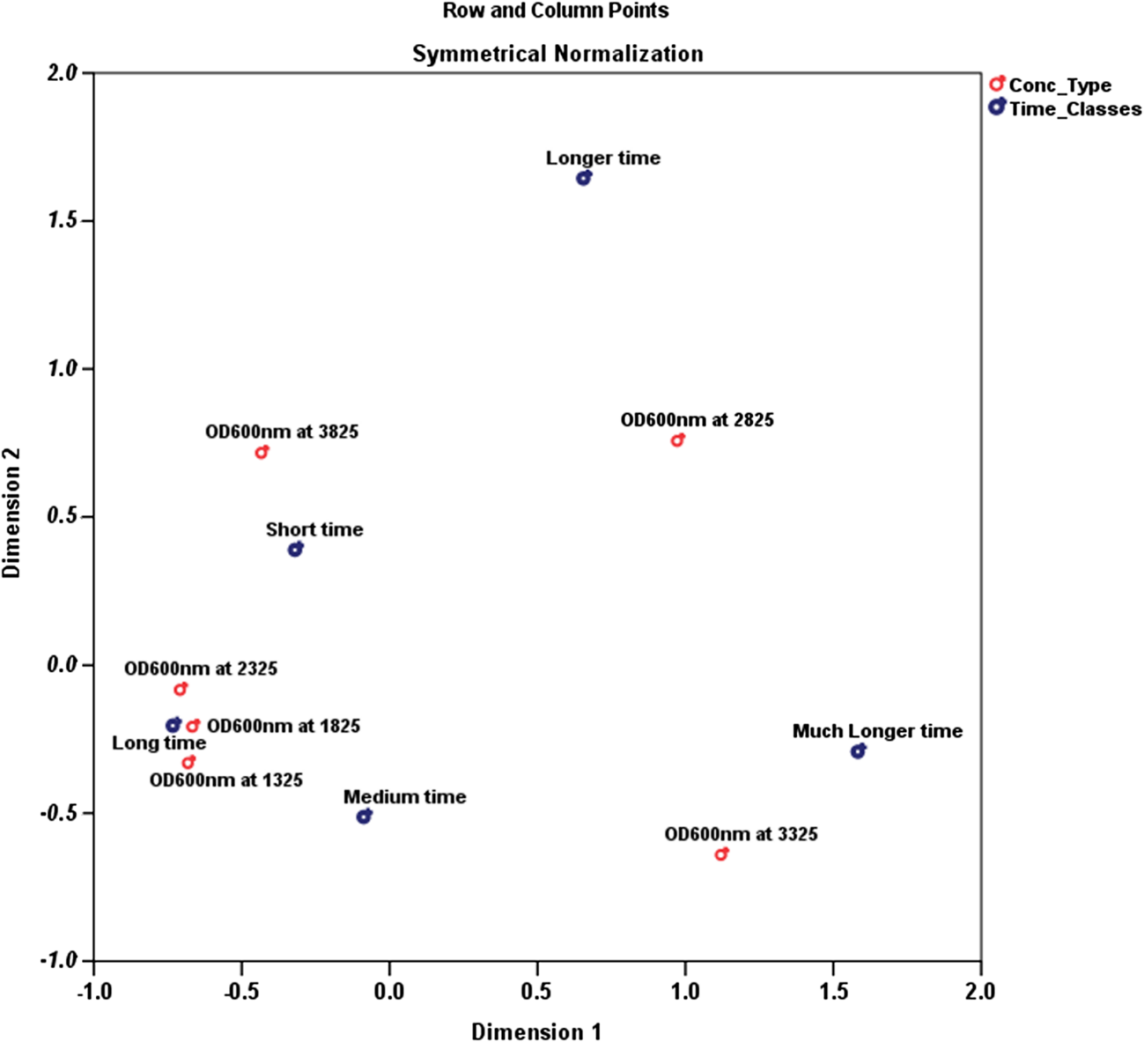
MCA plot of point clouds with hierarchical cluster analysis

The examples of the corresponding diagrams in which the existence of anomalous values is caused by the separation of one or more variables are shown in Fig. 7. Additionally, the separation of one or more samples in a certain direction suggests that the sample is abnormal for the relevant element. Furthermore, given their significant skewness toward the variable and lower skewness toward the nearby samples, these samples point to a greater possibility of anomaly of the relevant variable in the study. As a result, it suggests that OD600 nm at 3225 has a higher time than the other COD samples in both cluster plots. While OD600 nm at 3825 showed a shorter time of treatment. They are indicating that COD requires a much longer time than short-term treatment. However, OD600 nm across the six concentration scales showed a varied time of treatment but is much more significant from middle to long-time scales. COD removal in MFCs can be achieved through both short-time and long-time treatment. The optimal treatment time for COD removal varies depending on the specific conditions of the MFC system. For example, in the study by Naderia et al. (2022), short-time aeration was found to effectively reduce energy consumption and cost while ensuring effluent quality met standards.

In contrast, the study by Fazli et al. (2018), it was showed that changing the solid retention time (SRT) of the suspended biomass culture in MFCs significantly increased electricity production, indicating the relevance of SRT control in optimising MFC performance. Therefore, the time required for COD removal in MFCs can vary depending on the specific operating conditions and treatment strategies employed. The data presented in Table 4 reveals a decline in COD concentration as time progresses. This occurrence could be attributed to the consumption of organic substances in the leachate by the microorganisms within the system. Notably, the rate of COD elimination is most pronounced in the short-time class and subsequently diminishes over time. This observation implies that the microorganisms exhibit heightened activity during the initial stages of the treatment process. Upon examining the active margin section, it becomes evident that a substantial quantity of COD is removed over an extended duration. This finding suggests that the treatment system demonstrates efficacy in COD removal despite the gradual decrease in removal rate over time.

**Table 4:** Correspondence table of COD concentration and time

Conc_Type	Time_Classes					
	Short time	Medium time	Long time	Longer time	Much longer time	Active margin
OD600nm at 1325	4	3	6	0	0	13
OD600nm at 1825	5	3	6	0	0	14
OD600nm at 2325	5	2	6	0	0	13
OD600nm at 2825	5	1	0	2	6	14
OD600nm at 3325	1	4	2	1	9	17
OD600nm at 3825	3	2	5	2	0	12
Active margin	23	15	25	5	15	83

## Conclusions and recommendations

### Conclusions

In this comprehensive study on microbial fuel cells (MFCs) utilising landfill leachate as a substrate, we have elucidated several critical factors influencing the performance of MFCs in terms of power generation and wastewater treatment. The following key conclusions can be drawn:

1. The performance of MFCs is significantly affected by the concentration of chemical oxygen demand (COD) present in the leachate that enters the system. There is a positive correlation between higher concentrations of COD and improved power density, leading to better electricity output.
2. The pH level of the influent is a crucial component that significantly impacts the operation of MFCs. The researchers discovered that maintaining a pH level of around 6.88 was the most favourable condition for microbial activity and electron transfer, leading to increased current production. Deviations from this pH range adversely influenced performance.

This research contributes to our understanding of MFCs as promising bioelectrochemical devices for simultaneous wastewater treatment and renewable energy generation. It underscores the importance of carefully balancing operational parameters to maximise performance. Future endeavours should explore the scalability and long-term stability of MFCs under real-world conditions. With further research and development, MFCs have the potential to revolutionise sustainable water treatment and bioenergy production.

### Recommendations and further study

Building upon the insights gained from this study, there are several promising avenues for future research and practical applications of MFCs using landfill leachate as a substrate. These recommendations and prospects aim to advance the field and maximise the potential of MFC technology:

1. Future studies should focus on upscaling MFC systems to meet the demands of real-world wastewater treatment facilities. This includes optimising reactor design, electrode materials, and system configurations for larger volumes of landfill leachate.
2. Investigate the long-term stability and performance of MFCs under continuous operation. Understanding the microbial community dynamics, electrode fouling, and system degradation over extended periods is crucial for practical applications.
3. Explore innovative cathodic reactions to improve oxygen reduction kinetics and overall MFC efficiency. Alternative cathode materials and configurations may enhance power generation and extend electrode lifespan.
4. Investigate the potential of enriching electrogenic consortia specifically tailored for landfill leachate. This could involve selective enrichment techniques to cultivate bacteria with high electron transfer capabilities.
5. Consider hybrid systems that combine MFCs with other wastewater treatment technologies, such as activated sludge or anaerobic digestion. Synergistic effects can lead to more efficient organic matter removal and enhanced energy recovery.
6. Explore the potential for recovering valuable resources from treated landfill leachate, such as nutrients and metals. MFCs can serve as a platform for resource extraction while simultaneously treating wastewater. By pursuing these prospects and recommendations, the field of MFCs can advance towards more efficient, sustainable, and economically viable solutions for landfill leachate treatment while simultaneously contributing to renewable energy generation and resource recovery.

## Data Availability

The manuscript’s data is contained in the text.
